# Containing novel SARS-CoV-2 variants at source is possible with high-intensity sequencing

**DOI:** 10.1093/pnasnexus/pgac159

**Published:** 2022-08-19

**Authors:** Tobias S Brett, Pejman Rohani

**Affiliations:** Odum School of Ecology, University of Georgia, Athens, GA 30602, USA; Center for the Ecology of Infectious Diseases, University of Georgia, Athens, GA 30602, USA; Odum School of Ecology, University of Georgia, Athens, GA 30602, USA; Center for the Ecology of Infectious Diseases, University of Georgia, Athens, GA 30602, USA; Department of Infectious Diseases, College of Veterinary Medicine, University of Georgia, Athens, GA 30602, USA; Center for Influenza Disease and Emergence Research (CIDER), Athens, GA 30602, USA

**Keywords:** infectious diseases, mathematical modeling, dynamical systems

## Abstract

Throughout the COVID-19 pandemic, control of transmission has been repeatedly thwarted by the emergence of variants of concern (VOC) and their geographic spread. Key questions remain regarding effective means of minimizing the impact of VOC, in particular the feasibility of containing them at source, in light of global interconnectedness. By analysing a stochastic transmission model of COVID-19, we identify the appropriate monitoring requirements that make containment at source feasible. Specifically, precise risk assessment informed primarily by epidemiological indicators (e.g. accumulated hospitalization or mortality reports), is unlikely prior to VOC escape. Consequently, decision makers will need to make containment decisions without confident severity estimates. In contrast, successfully identifying and containing variants via genomic surveillance is realistic, provided sequence processing and dissemination is prompt.

Significance Statement As SARS-CoV-2 continues to circulate, the stochastic emergence of novel variants will pose a threat for the foreseeable future. Minimizing their impact requires identifying and containing each variant at source to halt onward circulation. Our results highlight that, due to the lag between exposure and death, identifying a novel variant using mortality data is unlikely before substantial spatial spread. On the other hand, our results suggest that by the start of 2022 multiple countries were sequencing sufficient infections to make successfully identifying and containing variants via genomic surveillance realistic, contingent on fast sequence processing, dissemination, and analysis times. As a consequence, while genomic surveillance makes early variant identification possible, it is not feasible to precisely quantify the threat posed before a proportionate containment response is needed.

## Introduction

The SARS-CoV-2 pandemic has been prolonged and exacerbated by the repeated emergence and global establishment of highly transmissible novel variants of concern (VOCs), including the Alpha, Delta, and Omicron variants. The continued societal impact of SARS-CoV-2 depends crucially on the frequency with which new variants emerge, their transmissibility, disease severity, and capacity to evade immunity, and our ability for early detection and prevention of geographic spread (1–5). The recent Omicron emergence and spread has highlighted the key bottlenecks in the containment of VOCs at source: the need for early detection and risk assessment of a novel virus via surveillance and diagnostics (including epidemiological, serological, and sequencing) and timely and proportionate public health efforts to cordon off the affected regions through coordinated travel restrictions (6, 7). Ultimately, the failure to prevent the subsequent spread of Omicron raises important questions regarding the effectiveness of early detection and travel restrictions at containing any future VOCs at source.

**Fig. 1.**
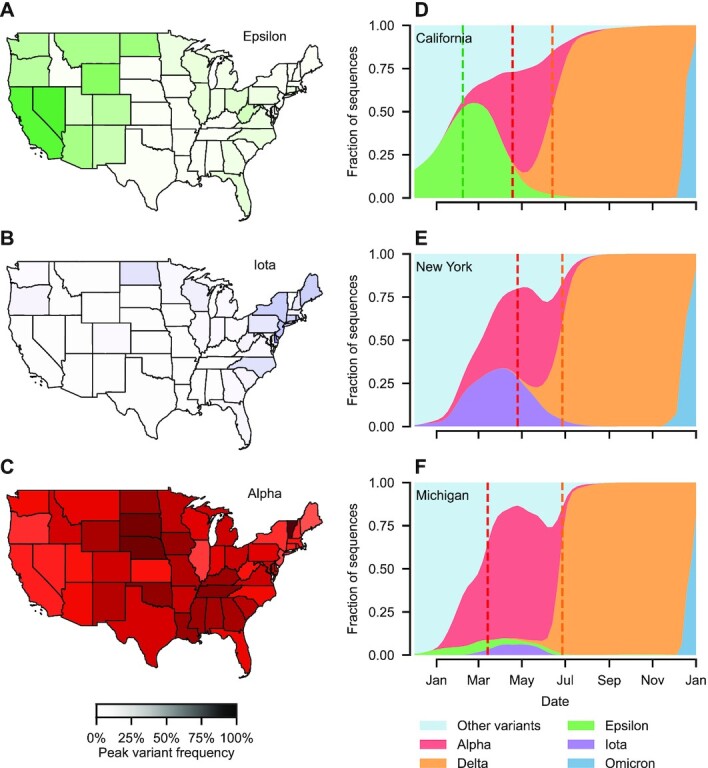
Uneven spatial spread of variants across the contiguous United States. (A to C) Prior to the Delta variant there was clear spatial variation in the frequency of different viral variants. The peak frequency of the Epsilon variant (first identified in California) was highest in western states (panel A), whereas the Iota variant (first identified in New York) was highest in the northeast. The invasive Alpha variant, which entered the United States after the emergence of both Epsilon and Iota, had the highest peak frequency in geographically central states in the south and mid-west. (D to F) The time variation in variant frequency for select states (one from each grouping identified above) illustrate these patterns in more depth. Both Epsilon and Iota appear to have enhanced transmissibility over pre-2021 variants (panels D and E). The higher frequency of these variants in west and east states reduced the competitive advantage of the Alpha variant and thereby slowed the Alpha wave. The subsequent invasion of Delta resulted in a selective sweep with near-total elimination of preexisting viral diversity in all states, preventing the eventual dominance of Alpha. Emerging in late November 2021, Omicron also completed a selective sweep and replaced Delta as the dominant strain.

To motivate the proceeding research, we describe successive waves of new emerging variants. Over the course of 2021, successive waves of Alpha (8–10), Delta (11, 1,2), and Omicron (13, 14) had swept across the world, triggering large outbreaks that many countries struggled to foresee and contain. In the United States, the spread of these and other variants exhibited clear spatio-temporal patterns (Fig. 1A), with successive waves of emergent variants replacing the previous. While the Delta and Omicron variants received much publicity on account of concerns regarding their increased transmissibility (14, 15) and potential for immune evasion (13, 16), other variants had previously emerged in the United States with contrasting spatio-temporal trajectories (Fig. 1). The Epsilon variant, first identified amidst a surge of cases in southern California in late 2020 October (17), briefly became the dominant variant in western states (Fig. 1A). Similarly, the Iota variant, which likely emerged in New York state around 2020 November (18), peaked highest primarily in northeastern states (Fig. 1B). For both variants, this geographic restriction suggests localized diffusion (to neighboring states) rather than long-range spread.

Conversely, the emergence of Alpha, Delta, and Omicron variants (all with external origins) was near-simultaneous across the country (e.g. Fig. 1C), suggesting a pivotal role for long-range spread. For instance, the increase in the frequency of Delta detections and subsequent near-fixation occurred synchronously across the United States, regardless of preexisting viral diversity (Fig. 1D to F). For each of these variants, increased surveillance and genetic sequencing led to their rapid identification, yet none were successfully contained either at source or at points of introduction, leading to widespread transmission across the United States.

To establish the conditions necessary for containing novel variants at source, mathematical models of emerging infectious diseases are insightful, as previously demonstrated in response to the 2003 SARS pandemic (19–27), among others. We formulate a spatial model of SARS-CoV-2 transmission that can accommodate the emergence of novel variants as well as multiple approaches to monitoring. Our model is a continuous-time Markov jump process (2,8,29) that captures the competition between variant exportation and detection (e.g. via random case sequencing) as its incidence in the source population grows. We derive analytical expressions for the probability of identifying the variant before it is exported. If control measures are then rapidly implemented, the novel variant could in principle be locally contained.

## Results

We first focus on the dynamics of epidemic spread in the absence of surveillance. Using our stochastic transmission model, we demonstrate how the speed of spatial variant escape is determined by the underlying epidemiological factors, including the variant reproductive number, *R*_0_, the mean infectious period, 1/γ, and the average immune protection in the destination (population *j*) against infection with the variant, *ϕ_j_*. Critically, the speed of variant spread is also determined by *c_i, j_*, which is the probability of contact between an infectious individual from the origin *i* and an individual in the destination *j*. This quantity can be estimated as the product of the *per capita* daily travel volume and the mean infectious period (see Section S2 of Supplementary Material).

**Fig. 2.**
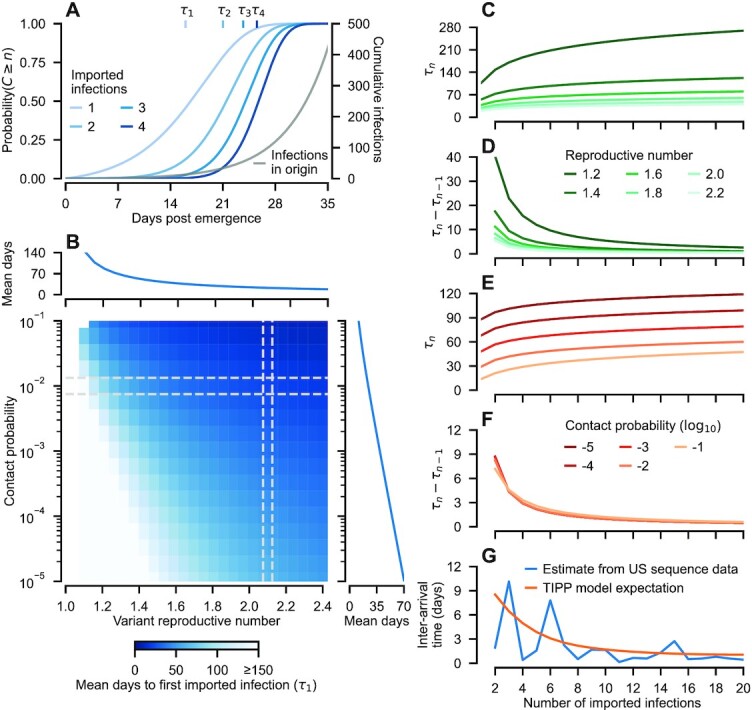
Probability and timings of exported variant infections. (A) The probability that at least *n* variant infections have been exported, for *n* = 1 to 5 from an exponentially growing outbreak in the origin location. The mean timing (in days postemergence, i.e. when *I_i_*(0) = 1) of the *n*th exported infection is indicated by *τ_n_* (see Supplementary Material Section S2, Eq. S23 for mathematical details). (B) Dependence of the mean day of the first exported infection, τ_1_, on the variant reproductive number, *R*_0_ and the inter-location contact probability, *c_i, j_*. For a given *R*_0_, τ_1_ has a roughly log-linear relationship with contact probability (see right inset). (C and D) At high reproductive numbers, successive importations rapidly follow the first (results shown for *c_i, j_* = 0.01). (E and F) Strikingly, the average interarrival time of successive importations, *τ_n_* − *τ*_*n* − 1_, is largely independent of the contact probability: while reducing it delays the first infection, it does not ameliorate the wave of subsequent introductions (results shown for *R*_0_ = 1.8). Consequently, if one importation is detected more will likely rapidly follow (*cf*. panel D). (G) Reconstruction of time between successive successful importations of the Alpha variant to the United States. Each importation sparked a unique chain of transmission large enough to be detected in a SARS-CoV-2 sequence data (30). We fitted our TIPP model to the estimated timing of Alpha variant importations, assuming they arrived from the United Kingdom (see the “Methods” section and Section S2 of Supplementary Material). For the results shown in panels A to F, we assumed *ϕ_i_* = *ϕ_j_* = 0, i.e. there is no prior immunity to the variant. Prior immunity in the origin (*ϕ_i_* > 0) translates into a smaller *α_i_*, whereas prior immunity in the destination (*ϕ_j_* > 0) is dynamically equivalent to a smaller *c_i, j_*.

Although our derivations hold more generally, we focus on the scenario where the incidence of the variant in the source population is growing exponentially at a rate *α_i_*, such that $I_i(t) = I_i(0) e^{\alpha _i t}$. Under these conditions, the number of imported cases in the destination, *Q_j_*(*t*), is a time-inhomogeneous Poisson process (TIPP), from which we derive expressions for (i) the expected time of the *n*th importation in *j*, $\tau _{n}^{(j)}$, and (ii) the expected interarrival times between successive introductions, $\tau ^{(j)}_n-\tau ^{(j)}_{n-1}$ (see Section S2 of Supplementary Material). This allows us to quantify the speed at which a novel variant spreads, and in particular how its spread is impeded by travel restrictions, which serve to decrease the contact probability, *c_i, j_* (Fig. 2). As variant incidence in the origin rises, so does the probability of imported infections in the destination (Fig. 2A). More importantly, these derivations also explain why travel restrictions have met with limited success (20–26). To illustrate this, we first point out that the variant reproductive number, *R*_0_, has a two-fold impact on the expected time to the first imported infection, $\tau ^{(j)}_1$, compared with the contact probability. A higher *R*_0_ (i) results in a faster accumulation of infections in the origin (i.e. a larger *α_i_*), each of which may spillover to the destination, and (ii) raises the spatial reproductive number, *R_i, j_* (see the “Methods” section). Of the two effects, the first dominates the second with *τ_n_* roughly proportional to 1/α_*i*_ and only logarithmically dependent on *R_i, j_*: $\tau _{n}^{(j)} \sim \frac{1}{\alpha _i} \ln (1+ b \alpha _i n/R_{i,j})$, where *b* is a constant determined by the epidemiological parameters (see Section S2 of Supplementary Material; Fig. 2B). In addition, because *R_i, j_*∝*c_i, j_*, there is also a log-linear relationship between contact probability and the mean time to the first imported infection (*n* = 1; Fig. 2B right insert).

To assess the prospects for containing a variant, it is also important to consider the expected interarrival time (Fig. 2C to F). This gives a measure of how extensive the public health response should be upon detection of an importation, e.g. whether other imported infections are likely to be missed. Decreasing *α_i_* not only increases the time until the first imported infection, it also lengthens the expected time between each imported infection (Fig. 2C and D). Conversely, while decreasing contact probability increases the time to the first imported infection (Fig. 2E), it has little impact on the time between successive importations (Fig. 2F). This means that once the first infection is imported, the time between successive infections is predominantly determined by the growth rate in the origin rather than the contact probability. For instance, if *α_i_* = 0.8 (equivalent to a 6-day doubling time), the twentieth imported infection will occur within one month of the first, regardless of the contact probability.

While previous modeling studies have focused on the difficulty in preventing any imported infections of a novel pathogen (19–27), our results highlight the practical difficulties in trying to prevent subsequent establishment of a pathogen, e.g. through contact tracing (31).

We compare our mathematical model with published results from a phylogenetic study, which estimated the number and timing of Alpha variant importations to the United States in winter 2020 to 2021 (30) (Fig. 2G). Each detected importation sparked a transmission chain sufficiently large to be detected in a data set of positive SARS-CoV-2 samples. Given uneven surveillance, the number of detected importations likely represents an underestimate (30). Using likelihood-based inference (see  Section S2 of Supplementary Material), we fitted our TIPP model to the median estimated importation time for each lineage detected in the phylogenetic study (blue line), assuming that they were exported from the United Kingdom (the Alpha variant population of origin) (Fig. 2G). Comparing these inference results with theoretical predictions derived from our model (Fig. 2F), we see in both cases a rapid early drop in the interarrival time, suggesting observed imports in the United States are consistent with exponential growth in the incidence of the variant in the United Kingdom. Our findings are robust to the estimated uncertainty in the timing of each Alpha importation (see Fig. S1 of Supplementary Material ).

The results presented in Fig. 2 show that an incomplete cessation of travel leads to a modest delay in the spread of a variant. Here, we demonstrate that such a delay can provide a crucial window for identification of a VOC and containment at source (see  Section S3 of Supplementary Material). By incorporating surveillance into our transmission model, we predict the outcome of the race between two competing processes: (i) successful detection of the variant in the origin, and (ii) the exportation of the virus to other regions.

**Fig. 3.**
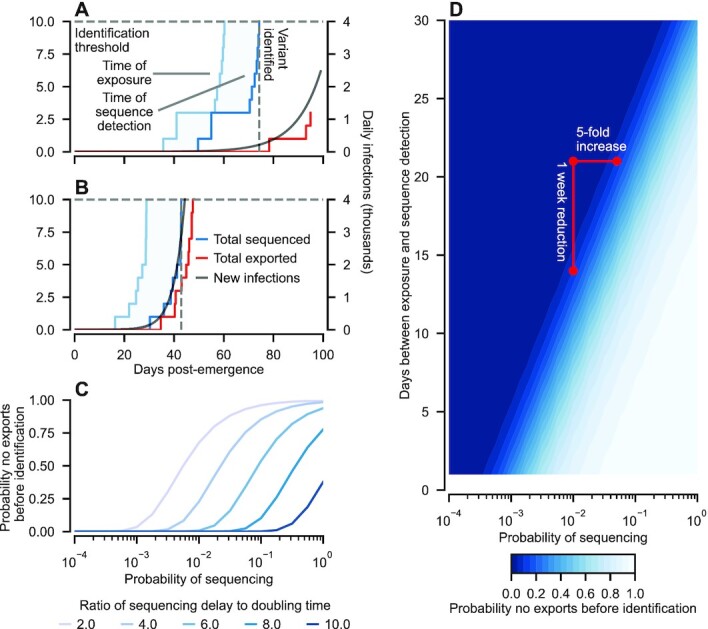
Impact of infection sequencing ratio and sequence processing delays on variant identification. (A) Sample realization of the variant emergence model. The observation delay (shaded region) is the lag between exposure (light blue) and sequencing results (dark blue). Once sufficient evidence exists via sequenced cases the variant is identified. Illustrative results shown here assume 10 sequences are necessary for identification, 1% of all infections are sequenced and the sequencing delay is 14 days. With a doubling time of 7 days, the variant is identified before any infections are exported. (B) For a variant with a 3-day doubling time, four infections are exported before the variant is identified. (C) As the ratio of sequencing delay to doubling time increases, it is necessary to sequence a greater proportion of infections to have the same probability of identifying the variant before exportation, *P*_0_. (D) A corollary is that reducing the number of days between exposure and sequencing results can have an impact comparable to substantial increases in sequencing. If the variant has a 3-day doubling time, then a 33% reduction in sequencing delay (e.g. from 3 to 2 weeks) has the same impact as a five fold-increase in infection sequencing probability. For all results shown in this figure, we used a contact probability *c_i, j_* = 10^−4^, assumed 10 sequences were necessary for variant identification, and that there was no prior immunity to the variant (*ϕ_i_* = *ϕ_j_* = 0).

We first examine the efficacy of variant identification based on the detection of atypical genetic sequences, before including epidemiological surveillance based on clusters of excess mortality. We assume that a proportion of infections are randomly sequenced, and that identification requires a threshold number of anomalous variant sequences (Fig. 3A and B). Whether the variant is identified before infections are exported depends on a range of factors, in particular the sequencing probability, the delay between exposure and sequencing results [for context, the median time to deposition on Global Initiative on Sharing All Influenza Data (GISAID) in December 2021 for SARS-CoV-2 sequences from the United States was around 21 days (32)], the contact probability, and the doubling time of variant infections in the origin (determined in turn by the reproductive number and serial interval). We find that the probability that no infections are exported before identification, *P*_0_, is acutely sensitive to the ratio of the sequencing delay to the doubling time (Fig. 3C). When the source epidemic is growing exponentially, the sequencing delay has the net effect of exponentially reducing the sampling probability, with the magnitude of reduction determined by the doubling time to sequencing delay ratio. This phenomenon is illustrated in Fig. 3D, highlighting that with our parameterization, a 1-week reduction in the delay between exposure and the dissemination of sequencing results has an identical impact on *P*_0_ to a five-fold increase in sequencing probability. In contexts where testing is constrained or costly, improvements in sequencing protocols can be decisive.

**Fig. 4.**
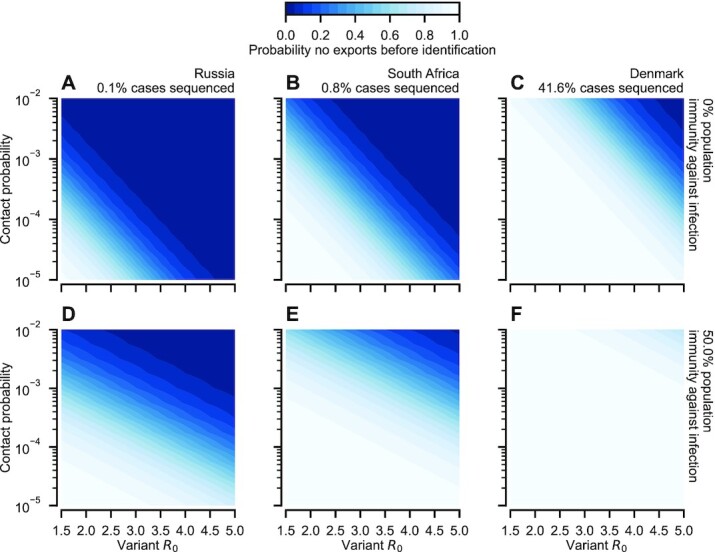
Prospects for containing a novel variant given a 2-week postexposure sequencing delay. (A to C) Heat maps showing the dependence of the probability of no infections are exported before the variant is identified on the variant *R*_0_ and the contact probability, assuming the variant is able to evade all immunity against infection in the population. Panels show three different countries, Russia (panel A), South Africa (panel B), and Denmark (panel C), selected based on the percentage of reported cases whose sequences were contributed to GISAID (32). (D to F) Same results as shown in panels A to C, but assuming the average population immunity against infection (through a combination of infection history and vaccination) is 50% in both populations. To provide a comparison across variant immune evasion, we present results in terms of the variant basic reproductive number, *R*_0_. Evaluating our results in the presence of nonpharmaceutical interventions amounts to scaling the *x*-axis. As in Fig. 3, we assumed 10 sequences were necessary for variant identification.

At the start of 2022, the proportion of reported cases whose sequences were disseminated publicly varied between countries by several orders of magnitude (32). While, due to infection under-reporting, these numbers represent an overestimate of the sequencing probability of an infection, they provide some indication of the prospects for identifying a variant emerging in each country. For instance, assuming a 2-week sequencing delay, containment of a novel variant capable of evading all immunity against infection, which emerged in Russia (with an estimated 0.1% case sequencing on GISAID) is practically impossible, even at low *R*_0_ and contact probability (Fig. 4A). Despite its higher sequencing probability, containment of such a variant in South Africa (0.8% case sequencing) is similarly unlikely (Fig. 4B). In contrast, in Denmark (41.6% case sequencing; Fig. 4C), identification before exportation is likely if the variant reproductive number is less than 3.5 and the contact probability is less than around 10^−4^ (comparable, for instance, to the average flux between London Heathrow and New York JFK in 2022 January; see Section S1 of Supplementary Material). If the average immunity in the population provides a 50% reduction to the probability of infection with the variant, prospects for containment in South Africa and Denmark improve markedly (Fig. 4E and F). Containment in Russia remains unlikely unless the variant possesses a basic reproductive number less than currently circulating variants (Fig. 4D).

**Fig. 5.**
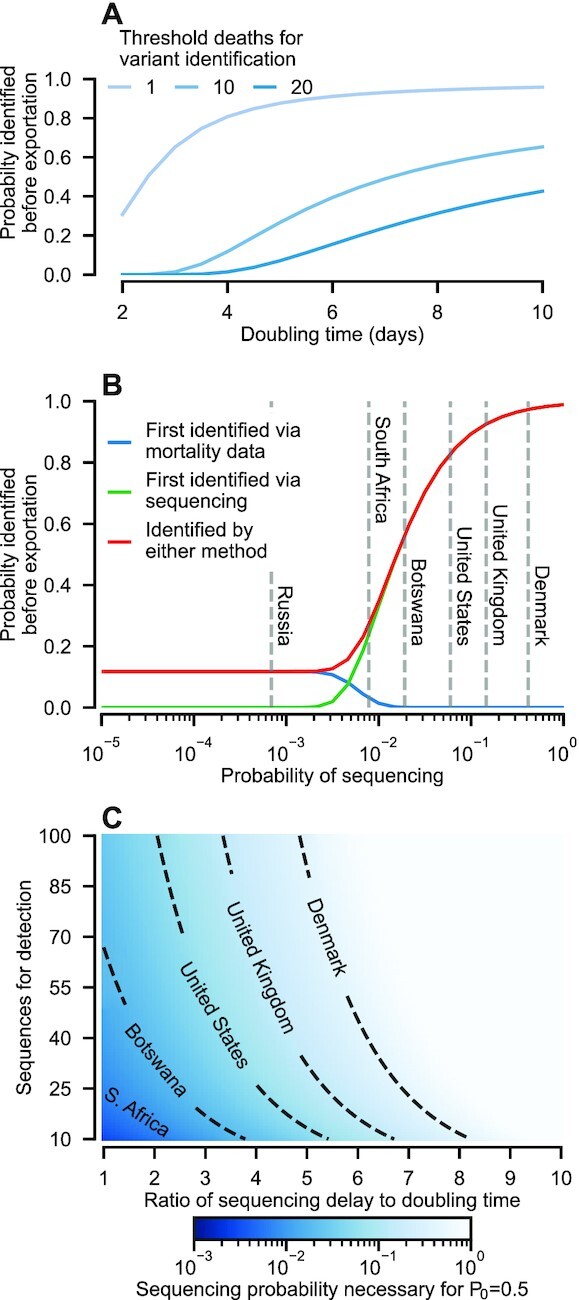
Challenges to detection of novel variants in mortality data. (A) Given the typically substantial lag between SARS-CoV-2 exposure and mortality, identification of a novel variant before it spreads to another population (connected with contact probability *c_i, j_* = 10^−4^) is unlikely unless the variant is identified after a small number of deaths (less than about 10) and the doubling time is greater than about 7 days. These findings hold even if the variant is capable of substantial mortality [results shown for infection fatality rate of 1%, comparable to early estimates of the original SARS-CoV-2 variant (33)]. (B) Our analytical results allow us to calculate the probability that the variant is first identified via monitoring of mortality data or sequence data. For both mortality and sequence monitoring we assume 10 observations (deaths and sequences, respectively) and a 2-week sequencing delay. For a doubling time of 4 days, with the same contact probability as panel A, the variant is more likely to be identified in sequence data first, if the probability of sequencing is greater than about 0.5%. (C) Sequencing probability necessary for a 50% chance of successfully identifying the variant (using either detection method) before any infections are exported. Results shown assume no prior immunity to the variant (*ϕ_i_* = *ϕ_j_* = 0).

To assess the relative reliability of detection methods that combine multiple data streams, we refined our model to account for variant identification via clusters of excess deaths alone (Fig. 5A) and together in combination with sequencing (Fig. 5B and C), and via vaccine breakthrough mortality (Fig. S2 of Supplementary Material).

To achieve this, we define a threshold number of variant-induced deaths (*d_M_*) and a threshold number of novel sequences (*d_S_*). We then derive (see Section S4 of Supplementary Material for details) an expression for the probability that a variant is first identified in mortality data, i.e. before: (i) any infections have been exported and (ii) *d_S_* infections are sequenced,

(1) $$\begin{eqnarray*}
P_M = \left(\frac{\kappa _M}{\kappa _M + \kappa _E}\right)^{d_M} \sum _{z=0}^{d_S-1}f\left(z; d_M, \frac{\kappa _S}{\kappa _M + \kappa _S + \kappa _E}\right),
\end{eqnarray*}
$$

where *f*(*z; n, p*) is the probability mass function of the negative binomial distribution and *κ_M_*, *κ_S_*, and *κ_E_* are the rates at which infections are fatal, sequenced, and exported, respectively. Both *κ_M_* and *κ_S_* have the same functional structure, *κ_M_* = *p_M_m_M_*(α_*i*_)*R_i, i_* and *κ_S_* = *p_S_m_S_*(α_*i*_)*R_i, i_*, where *p_M_* and *p_S_* are the probabilities an infection is included in mortality data and sequenced data respectively, while *κ_E_* = *R_i, j_*. The two discounting factors, *m_M_*(*α_i_*) and *m_S_*(*α_i_*), are determined by the epidemic growth rate and the lags between exposure and mortality and between exposure and sequencing results, respectively (see Section S4 and Eq. S38 for details of Supplementary Material). The mathematical structure of Eq. (1) stems from *P_M_* being a product of two probabilities: (i) a geometric distribution $\left(\frac{\kappa _M}{\kappa _M + \kappa _E}\right)^{d_M}$ that corresponds to the probability the variant is identified in mortality data before any infections are exported, and (ii) the probability that the variant is not first identified in sequence data, $\sum _{z=0}^{d_S-1}f(z; n, p)$. The probability that the variant is first identified in sequencing data, *P_S_*, is found by exchanging the subscripts *M* and *S* in Eq. (1). The total probability that the variant is identified before any cases are exported is given by *P*_0_ = *P_M_* + *P_S_*.

Analysis of Eq. (1) reveals that identification of a new variant before infections are exported based exclusively on mortality data is only plausible if the doubling time is at least 1 week, and the threshold number of deaths necessary for identification is less than around 20 (Fig. 5A). These constraints stem from the substantial delay between symptom onset and mortality, which is ∼26 days for COVID-19 (34).

Eq. (1) also reveals that, in general, variant identification occurs first in sequence data, unless sequencing efforts are sparse (Fig. 5B). For instance, assuming variant identification requires (i) either 10 deaths or the detection of 10 anomalous sequences, (ii) a 4-day doubling time, and (iii) a 2-week sequencing delay then identification is only more likely to occur first through mortality data if fewer than 0.5% of infections are sequenced. Estimates of sequencing efforts from early 2022 indicate that a number of countries have exceeded this threshold (Fig. 5B). Thus, taken in combination, our results (Figs. 3 to 5) overwhelmingly favor using sequencing as the monitoring strategy if the aim is to contain the spread of novel variants. Further, our findings have relevance to decisions on the necessary epidemiologic and genomic surveillance for a given risk-tolerance of missing an emerging VOC. Specifically, we calculated the threshold sequencing effort necessary for VOC containment at source for a given risk of identification failure (Eq. S46 in Section S3 of Supplementary Material; Fig. 5C). For instance, with its sequencing probability at time of writing, Denmark will identify the VOC with greater than 50% chance of success before spread even with a high sequence count threshold, provided the ratio of sequencing delay to doubling time is less than around 6 (for an Omicron-like variant with a 3- day doubling time, this corresponds to an 18-day sequencing delay).

Finally, using vaccine breakthrough deaths to identify a novel variant presents additional challenges (see Section S3 of Supplementary Material). As shown in Fig. S2 of Supplementary Material, incomplete vaccine coverage results in a pool of unvaccinated who are unobservable by the monitoring protocol. Transmission in this group can lead to substantial incidence and elevated exportation risk, without increasing detection prospects (Fig. S2D of Supplementary Material). Therefore, although such a monitoring scheme is tailored to identify the most high-risk variants, it stands the least chance of success.

## Discussion

Given that eradication of SARS-CoV-2 is unlikely, the sporadic emergence of novel variants (Fig. 1) will pose a threat for the foreseeable future (35). The spectre of a repeating cycle of variant emergence and subsequent disruptive public health measures has proven to be disheartening and exacerbated pandemic fatigue (36). While early variant detection is clearly an ambitious goal, the benefits—in terms of reducing loss of life as well as social and economic disruption—are enormous. Minimizing the impacts of a variant requires identifying and containing it at source as early as possible to halt onward circulation. As demonstrated by the response to the emergence of Omicron, knee-jerk and inconsistent travel restrictions (as imposed by multiple countries) do little to curtail global spread (37).

Travel restrictions employed by various countries were successful in the early stages of the pandemic. In particular, nations such as China, New Zealand, and Australia imposed severe travel restrictions, seeking to effectively epidemiologically decouple themselves from the rest of the world and thereby prevent viral introductions (38). For countries attempting to halt all endogenous disease transmission, popularly called a “zero COVID” strategy, questions remain regarding whether and how long such isolation policies must be maintained, the degree to which travel restrictions can be relaxed while remaining successful, and their viability in the face of emerging highly transmissible variants (39). Our results reinforce the conclusion that any reductions in travel short of quarantining all arrivals until they are confirmed uninfected is unlikely to prevent importation (Fig. 2)—consistent with studies carried out in response to the 2003 SARS-CoV-1 outbreak (20–26). Mathematically, this results from a log-linear relationship between the reduction in travel and the expected time of the first imported infection (40) (see Section S2 of Supplementary Material) such that a 90% reduction in travel flow may delay the first imported infection by 2 weeks, while a 99% reduction might only delay it by 4 weeks. In addition, our results highlight a key challenge highly transmissible variants pose to containment efforts: The shorter the origin doubling time, the shorter the expected time between successive importations in the destination (Fig. 2F and  G; see Section S2 of Supplementary Material). Consequently, reactive travel restrictions imposed after the first detected imported case will be too late.

The crucial quantity in determining the prospects of containing a variant is the ratio of detection delay to variant doubling time (Fig. 3). Indeed, increases in the timeliness of sequence dissemination can result in comparable gains to substantial improvements in sequencing coverage (Fig. 3D). This provides information for prioritizing surveillance efforts and protocols given constrained resources. Given the dependence of the ability to identify variants before exportation on key epidemiological parameters (see Section S3 of Supplementary Material), we have demonstrated that increasing coverage with vaccines that provide at least partial protection against variant infection will always slow their spread (Fig. S2 of Supplemenatry Materials). Furthermore, vaccination in the source population has a substantially greater impact than vaccination in the recipient population, due to the reduction in the ratio of detection delay to doubling time (see Fig. 3C).

Perhaps counter-intuitively, our results suggest that it might be easier to contain a variant that emerges within a closely monitored country than to prevent the importation and subsequent spread of one which emerges in another country with poor monitoring. If monitoring efforts are both comprehensive and highly spatially resolved, then the detection of the variant is likely to precede substantial spread beyond highly connected immediately neighboring communities (as shown in Fig. 4F). On the other hand, if the variant emerges in a country with poor monitoring then exportation is likely prior to detection. Due to exponential growth in the origin, outbreaks will likely be seeded by travelers across the destination country before travel restrictions can be imposed, as witnessed by the spread of Alpha, Delta, and Omicron in the United States (shown in Fig. 1) and globally.

Throughout our analysis, we focused on the probability of identifying a variant before any infections are imported—a conservative measure of the prospects of containing a variant given that the transmission chain of an imported infection might terminate early on due to stochastic effects. Individual variation in transmission rates (“superspreading”) have been shown to play a key role in whether an imported infection sparks widespread transmission (the “invasion probability”), with more overdispersed offspring distributions resulting in lower invasion probabilities (41). The exact extent of overdispersion for SARS-CoV-2 is uncertain, and is likely context-dependent, with estimates of the dispersion parameter, *k*, ranging from 0.1 to 0.58 (42). To account for the potential impact of superspreading, our modeling approach can readily be adapted by conditioning only on successful invasion. To achieve this, we would multiply *κ_E_* in Eqs. (1) and (11) by the invasion probability corresponding to the values of *R*_0_ and *k*.

In this paper, we have explored two different methods for identifying novel variants: by random sequencing of confirmed cases and by monitoring mortality data. Other methods of identification have been proposed, for instance by sequencing SARS-CoV-2 samples from wastewater (43). In principle, our modeling approach could be adapted to wastewater monitoring, provided data exist that can reasonably parameterize the per infection probability of a sample being detected in wastewater, and the delay between exposure and sample detection. A novel variant could also be identified through a sudden spike in SARS-CoV-2 incidence (unclassified by strain), which we consider briefly in Section S5 of Supplementary Material. As the delay between exposure and case confirmation of infection (e.g. via PCR testing) is typically shorter than the delay before either sequencing results or mortality, it has an advantage as a variant identification method. We did not focus further on this particular identification method due to its limitations, in particular its lack of specificity; a spike in cases may be driven by a range of mechanisms unrelated to variant emergence.

Our analytical results explain that, due to the lag between exposure and death, the variant is most likely to be identified first in sequence data (Fig. 5B). An important corollary is that it is not feasible to quantify precisely the risk of a novel variant before a proportionate response is needed (Fig. 5A). Given the humanitarian, societal, and economic impacts, a public framework on how to make containment decisions given incomplete epidemiological information is urgently required. Without prior agreement, any future variant emergence events will almost certainly pose the same antagonisms and failures witnessed with Omicron.

## Methods

### Data

Data on the variant membership metadata for United States and England for SARS-CoV-2 sequences were downloaded from nextstrain.org on 2022 February 4 (44). Sequences were subsampled for each state from the dataset generated by the CDC’s SPHERES consortium (https://www.cdc.gov/coronavirus/2019-ncov/covid-data/spheres.html). Variant membership data for sequences from England were also downloaded from nextstrain.org, subsampled from the open dataset sourced from GenBank (45). Epidemiological data for England (including time series of daily confirmed cases) are collated and made publicly available by the UK government (https://coronavirus.data.gov.uk/). Data for the percentage of reported cases that were sequenced and shared in the previous 90 days were downloaded from GISAID on 2021 December 16. Alpha variant incidence in the United Kingdom was reconstructed by weighting the daily confirmed cases by the England Alpha variant frequency estimate (see below).

### Variant frequency estimates

Variant frequencies through time were estimated from the sequence metadata for each US state and England individually and required two steps. First, for each variant *a*, the time-varying scaled prevalence, *K_a_*(*t*), was found using kernel density estimation (KDE) on the time series of sequence sampling dates for all sequences of the variant in the given location. Second, the variant frequency was calculated from the scaled prevalances, *f_a_*(*t*) = *w_a_K_a_*(*t*)/∑_*a*_*w_a_K_a_*(*t*), where *w_a_* is the total number of samples for variant *a* from the location.

### Stochastic transmission model

At the core of our analysis is a stochastic spatial Susceptible–Exposed–Infected–Recovered–Vaccinated (SEIRV) model that models the spread of a variant from its point of origin to other locations. To account for demographic stochasticity (crucially important during the early stages of the emergence of a novel variant), we formulated our model as a continuous-time discrete-state space Markov jump process. Following the standard SEIR model scheme (46), the population at each spatial location *j* is subdivided into five compartments, with *S_j_*, *E_j_*, *I_j_*, *R_j_*, and *V_j_* denoting the number of individuals who are susceptible, exposed, infected, recovered, and unexposed vaccinated, respectively. Throughout this paper, we assumed a short latent period 1/σ = 2 days [see (47)] and infectious period 1/γ = 7 days [see (48)]. We assumed that the protection against infection with the variant conferred by vaccination and prior infection is ε_*V*_ and ε_*R*_, respectively. Following Kurtz (49), the dynamics can be encoded in a system of stochastic equations. In the interests of space, the full stochastic model is given in Section S1 of Supplementary Material. If the incidence of the variant is high in location *j*, then the stochastic dynamics can be approximated by a system of ordinary differential equations (49),

(2) $$\begin{eqnarray*}
\frac{dS_{j}}{dt} = - \lambda _{j}(t) S_j,
\end{eqnarray*}
$$

(3) $$\begin{eqnarray*}
\frac{dE_{j}}{dt} = \lambda _{j}(t)(1 -\phi _j(t))N_j - \sigma E_{j},
\end{eqnarray*}
$$

(4) $$\begin{eqnarray*}
\frac{dI_{j}}{dt} = \sigma E_{j} - \gamma I_{j},
\end{eqnarray*}
$$

(5) $$\begin{eqnarray*}
\frac{dR_{j}}{dt} = - (1-\epsilon _R) \lambda _{j}(t)R_j + \gamma I_j,
\end{eqnarray*}
$$

(6) $$\begin{eqnarray*}
\frac{dV_{j}}{dt} = - (1-\epsilon _V) \lambda _{j}(t)V_j,
\end{eqnarray*}
$$

where λ_*j*_(*t*) is force of infection experienced by susceptible individuals in location *j*, given by

(7) $$\begin{eqnarray*}
\lambda _{j}(t) = \sum _i \frac{\beta c_{i, j}}{N_j} I_{i}(t),
\end{eqnarray*}
$$

with β denoting the transmissibility of the variant and *c_i, j_* the spatial contact probability—the proportion of contacts infectious individuals in *i* have with individuals in *j*. For our two-population network, the contact probability *c_i, i_* is given by *c_i, i_* = 1 − *c_i, j_*. This parameterization ensures that the basic reproductive number of the variant, *R*_0_, is *R*_0_ = β/γ regardless of *c_i, j_*. We have also introduced *ϕ_j_*(*t*), the average immune protection in population *j* to infection with the variant:

(8) $$\begin{eqnarray*}
\phi _j(t) = 1 - \frac{S_j(t) + (1-\epsilon _V) V_j(t) + (1-\epsilon _R) R_j(t)}{N_j}.
\end{eqnarray*}
$$

As our analysis depends only on the composite quantity *ϕ_j_*, we do not consider explicit values for the underlying model compartments (*S_j_*, *V_j_*, and *R_j_*) and protection strength parameters (ϵ_*V*_ and ϵ_*R*_).

### Modeling spatial spread of variants

Starting from a spatial stochastic transmission model, we derived analytical results for the statistics of successive viral exportation events. Our focus is on the early stages of the spread of a variant from its origin location, *i*. The cumulative number of imports in the destination, *Q_j_*(*t*), is given by a counting process (29),

(9) $$\begin{eqnarray*}
Q_j(t) = Y_j^Q\left(\int _0^t \gamma R_0 c_{i,j} (1-\phi _j(s))I_{i}(s)ds\right),
\end{eqnarray*}
$$

where $Y_j^Q\left(u\right)$ is a Poisson process and *I_i_*(*t*) is the number of infectious individuals in the origin at time *t*. To contextualize Eq. (9), the expected cumulative number of imported infections, $\mathbb {E}[Q_j(t)] = \int _0^t \gamma R_0 c_{i,j} (1-\phi _j(s))I_{i}(s)ds$, is equal to the integral of the first term on the right-hand side of Eq. (3) when secondary transmission in *j* is neglected. The expected number of importations in *j* caused by an infectious individual in *i, R_i, j_*, is given by *R_i, j_* = *R*_0_*c_i, j_*(1 − *ϕ_j_*). Using Eq. (9), we derived probabilistic expressions for the timings of imported cases, allowing us to (i) perform statistical inference on importation data for arbitrary epidemic dynamics in the origin, and (ii) calculate the expected interarrival time between imported cases as a function of *R*_0_ and *c_i, j_* in the case of exponential growth in variant incidence in the source population, $I_i(t) = I_i(0) e^{\alpha _i t}$ with rate *α_i_* ≈ γ(*R_i, i_* − 1), where *R_i, i_* = *R*_0_(1 − *c_i, j_*)(1 − *ϕ_i_*) is the local reproductive number in *i* (see Section S2 of Supplementary Material for full mathematical details). For clarity, at places results are presented in terms of the doubling time, *t*_2_ = ln (2)/*α_i_*.

### Modeling the identification of variants

We derived a mathematical expression for the probability of making *d* observations of the variant in the origin location before it causes *n* imported infections in location *j*. After *d_O_* cases of the variant are detected, the variant is identified and potentially contained (see Section S3 of Supplementary Material). We first present the derivation of our results for a general monitoring process, and then consider two specific data streams: random infection sequencing data and mortality data.

We assume that the monitoring effort captures a fraction of variant infections, *p_O_*. As with the number of exported infections, the number of detected variant infections, *D_i_*(*t*), is given by a counting process,

(10) $$\begin{eqnarray*}
D_{i}(t) = Y_{i}^{ D}\left[\int _0^t \!ds\int _0^{s}\!ds^{\prime }\, p_{ O} \gamma R_{i,i} K(s-s^{\prime }) I_{i}(s^{\prime })\right],
\end{eqnarray*}
$$

where $Y_{i}^{D}(u)$ is a Poisson process, *K*(*s* − *s*′) is the distribution of lags between infection and observation, and *R_i, i_* is the local reproductive number defined above.

Using Eqs. (9) and (10), we derived expressions for the probability that the variant is identified before any infections are exported. If the incidence is growing exponentially in the origin, $I_i(t) = I_i(0) e^{\alpha _i t}$, then the number of infections exported before the variant is identified follows a negative binomial distribution with probability mass function *f*(*n; d_O_*, *q*), where *q* is given by

(11) $$\begin{eqnarray*}
q = \frac{{\kappa _O}}{{\kappa _O + \kappa _E}},
\end{eqnarray*}
$$

with *κ_O_* = *p_O_m*(*α_i_*)*R_i, i_* and *κ_E_* = *R_i, j_* being the rates at which infections are detected and exported, respectively. The discounting factor *m*(*α_i_*) is determined by the detection delay distribution and the origin epidemic growth rate, *α_i_*. The probability the variant is identified before any cases are exported is given by $P_0 = f(0; d_O, q) = q^d_O$. For details on the derivation and parameterization of the sequencing data and mortality data monitoring strategies considered in the paper (see Section S3 of Supplementary Material).

### Simulation algorithm

Numerical simulations of the SARS-CoV-2 transmission model were performed using the Euler Multinomial algorithm (50). The algorithm simulates a discrete time approximation to the continuous time stochastic SEIR model (analogous to the Euler forward algorithm for solving ordinary differential equations), appropriate for efficient simulation of stochastic dynamics in large populations. Simulations were performed using a timestep of 0.01 weeks.

## Supplementary Material

pgac159_Supplemental_Files

## Data Availability

The data and source code underlying this article are available in the zenodo repository 10.5281/zenodo.7026697, and can be accessed with the url https://doi.org/10.5281/zenodo.4000983.
