# Supplementary material for: Containing novel SARS-CoV-2 variants at source is possible with high-intensity sequencing
Source: PNAS Nexus. 2022 Aug 19;1(4):pgac159. doi: 10.1093/pnasnexus/pgac159 (PMC9465520; doi:10.1093/pnasnexus/pgac159)
Supplement: pgac159_Supplemental_Files [file pgac159_supplemental_files.zip › PNASNEXUS-PNASNEXUS-2022-00266-T-s02.pdf]

Supplementary Information for

Containing novel SARS-Cov-2 variants at source is possible with high intensity sequencing

Tobias S. Brett, Pejman Rohani

Tobias S Brett
E-mail: tsbrett@uga.edu

This PDF file includes:

Supplementary text
Figs. S1 to S2
SI References

Supporting Information Text

S1. Stochastic transmission model

At the core of our analysis is a stochastic spatial Susceptible-Exposed-Infected-Recovered-Vaccinated (SEIRV) model that models the spread of a variant from its point of origin. To account for demographic stochasticity (crucially important during the early stages of the emergence of a novel variant), we formulated our model as a continuous-time discrete-state space Markov jump process. We assumed the virus circulates on a spatial network of M spatial locations (indexed $j = 1, \dots M$) each with population size N_j. We focus on the specific case where $M = 2$, with one population being the origin and the other the destination. Following the standard SEIR model scheme (1), the population at each spatial location j is sub-divided into five compartments, with S_j, E_j, I_j, R_j and V_j denoting the number of individuals who are susceptible, exposed, infectious, recovered and unexposed vaccinated respectively. We assumed a short latent period $1/\sigma = 2$ days (see (2)) and infectious period $1/\gamma = 7$ days (see (3)). We assumed that the protection against infection with the variant conferred by vaccination and prior infection is ε_V and ε_R, respectively. Protection strength ranged from zero (no protection against infection with variant) to 1 (perfect protection against infection). Following Kurtz (4), the dynamics can be be encoded in a system of stochastic equations (which are more suited to our purposes than the more common master equation formalism (5)),

$$S_j(t) = S_j(0) - Y_j^S\left(\int_0^t ds\, \lambda_j(s) S_j(s)\right), \quad \text{[S1]}$$

$$E_j(t) = E_j(0) + Y_j^S\left(\int_0^t ds\, \lambda_j(s) S_j(s)\right) + Y_j^R\left(\int_0^t ds\, (1-\epsilon_R)\lambda_j(s) R_j(s)\right)$$
$$+ Y_j^V\left(\int_0^t ds\, (1-\epsilon_V)\lambda_j(s) V_j(s)\right) - Y_j^E\left(\int_0^t ds\, \sigma E_j^S(s)\right), \quad \text{[S2]}$$

$$I_j(t) = I_j(0) + Y_j^E\left(\int_0^t ds\, \sigma E_j(s)\right) - Y_j^I\left(\int_0^t ds\, \gamma I_j(s)\right), \quad \text{[S3]}$$

$$R_j(t) = R_j(0) + Y_j^I\left(\int_0^t ds\, \gamma I_j(s)\right) - Y_j^R\left(\int_0^t ds\, (1-\epsilon_R)\lambda_j(s) R_j(s)\right), \quad \text{[S4]}$$

$$V_j(t) = V_j(0) - Y_j^V\left(\int_0^t ds\, (1-\epsilon_V)\lambda_j(s) V_j(s)\right), \quad \text{[S5]}$$

for $j = 1, \dots, M$. Each $Y_j^\alpha(\cdot)$ is a counting process, with expectation $\mathbb{E}[Y_j^\alpha(u)] = u$ (6). The processes $Y_j^S(\cdot)$, $Y_j^R(\cdot)$, $Y_j^V(\cdot)$ are the infection of susceptible, recovered and vaccinated individuals in location j, the process $Y_j^E(\cdot)$ is individuals moving from the exposed (latent) class to the infectious class and $Y_j^I(\cdot)$ is the movement of infectious from the infectious class to the recovered/removed class.

The force of infection experienced by susceptible individual in location j is given by

$$\lambda_j(t) = \sum_i \frac{\beta c_{i,j}}{N_j} I_i(t), \quad \text{[S6]}$$

where β is the transmissibility the variant and $c_{i,j}$ is the spatial contact probability: The proportion of contacts infectious individuals in i have with individuals in j. For our model of variant spread, $c_{i,j}$ can be estimated from the proportion of the infectious period an individual spends in the origin versus the destination. If the *per capita* probability of travelling from i to j per day, $m_{i,j}$, is small (as is the case for international travel) then $c_{i,j} \approx m_{i,j}/\gamma$. For instance, in January 2022 a total of 219199 passengers travelled between London Heathrow airport and New York JFK airport – approximately 3500 passengers per day in each direction. Dividing by the population size of the UK (around 67 million (7)) gives $m_{i.j} \approx 5.3 \times 10^{-5}$ and $c_{i,j} \approx 3.7 \times 10^{-4}$. For our two population network, the contact probability $c_{i,i}$ is given by $c_{i,i} = 1 - c_{i,j}$. This parameterisation ensures that the basic reproductive number of the variant, R_0, is $R_0 = \beta/\gamma$ regardless of $c_{i,j}$.

In principle vaccination (i.e. movement of individuals from the S class to the V class) could be included in the model, however for our purposes it is unnecessary (see below). Modifications to the model to account for case sequencing and mortality are considered below.

At large population sizes (4) the stochastic dynamics can be approximated by a system of ordinary differential equations,

$$\frac{dS_j}{dt} = -\lambda_j(t)S_j, \quad \text{[S7]}$$
$$\frac{dE_j}{dt} = \lambda_j(t)(1-\phi_j(t))N_j - \sigma E_j, \quad \text{[S8]}$$
$$\frac{dI_j}{dt} = \sigma E_j - \gamma I_j, \quad \text{[S9]}$$
$$\frac{dR_j}{dt} = -(1-\epsilon_R)\lambda_j(t)R_j + \gamma I_j, \quad \text{[S10]}$$
$$\frac{dV_j}{dt} = -(1-\epsilon_V)\lambda_j(t)V_j, \quad \text{[S11]}$$

where we have introduced $\phi_j(t)$, the average immune protection in the population to infection with the variant:

$$\phi_j(t) = 1 - \frac{S_j + (1-\epsilon_V)V_j + (1-\epsilon_R)R_j}{N_j}. \quad \text{[S12]}$$

S2. Modelling spatial spread of variants

A. General case. By approximating our stochastic transmission model, we were able to derive analytical results for the statistics of successive viral exportation events. We focus on the early stages of the spread of a variant from its origin location, i. From Eq. S2, the cumulative number of exposed individuals in location j (the destination) is given by

$$Q_j(t) = Y_j^S\left(\int_0^t ds\,\lambda_j(s)S_j(s)\right) + Y_j^R\left(\int_0^t ds\,(1-\epsilon_R)\lambda_j(s)R_j(s)\right) + Y_j^V\left(\int_0^t ds\,(1-\epsilon_V)\lambda_j(s)V_j(s)\right). \quad \text{[S13]}$$

We can superimpose the three counting processes in Eq. S13 to write $Q_j(t)$ in terms of a single counting process,

$$Q_j(t) = Y_j^Q\left(\int_0^t ds\,\lambda_j(s)(1-\phi_j(s))N_j\right), \quad \text{[S14]}$$

where we have also used the definition of $\phi_j(t)$, the average immunity, Eq. S12. In general, Eq. S14 counts the total number of exposed individuals in the destination. We can limit it to only counting importations by excluding local transmission in j from the force of infection, i.e. fixing $\lambda_j(t) = \beta c_{i,j} I_i(t)/N_j$.

Furthermore, as we are interested in the early dynamics of spatial spread, infections in the destination are initially small relative to the origin. We therefore can assume there is negligible transmission back to location i over the period of interest, and that the origin transmission dynamics are independent of those in the destination. We also assume that over the timescale of interest (typically of order 1-2 months), there is little change in $\phi_j(t)$, and therefore take it to be constant. Consequently, conditional on a realisation of the dynamics in the origin, Eq. S14 corresponds to a time-inhomogeneous Poisson process (TIPP), $Q_j(t) = Y_j^Q\left(\int_0^t ds\,h_j(s)\right)$, with hazard function

$$h_j(t) = \beta c_{i,j}(1-\phi_j)I_i(t), \quad \text{[S15]}$$

and cumulative hazard $\Lambda_j(t) = \int_0^t ds\,h_j(s)$. As $h_j(t) \geq 0$ for all t, $\Lambda_j(t)$ is a monotonic increasing function of t. Later we focus on a particular case (exponentially growing epidemic in the origin), but for now we keep the analysis general.

The probability that, at time t, the number of imported infections, $Q_j(t)$ is equal to n, is given by

$$P_n^{(j)}(t) = \frac{\Lambda_j(t)^n}{n!}e^{-\Lambda_j(t)}. \quad \text{[S16]}$$

Furthermore, the set of $P_n^{(j)}(t)$ satisfy a system of ODEs known as the master equation (5),

$$\frac{dP_n^{(j)}(t)}{dt} = h_j(t)\left(P_{n-1}^{(j)}(t) - P_n^{(j)}(t)\right) \text{ for } n = 1,2,3,\ldots,$$
$$\frac{dP_0^{(j)}(t)}{dt} = -h_j(t)P_0^{(j)}(t). \quad \text{[S17]}$$

Once in this form we can use standard techniques from the theory of first-passage processes (5). The probability that the n-th importation arrives at time $T_n^{(j)}$ is given by

$$f_n^{(j)}(t) = h_j(t)P_{n-1}^{(j)}(t) \text{ for } n = 1,2,3,\ldots, \quad \text{[S18]}$$

and subsequently the expected time of the n-th importation in j is given by

$$\tau_n^{(j)} = \int_0^\infty dt\, t f_n^{(j)}(t) \\ = \int_0^\infty dt\, t h_j(t) P_{n-1}^{(j)}(t). \tag{S19}$$

By re-arranging Eq. S17, we can substitute for $P_{n-1}^{(j)}(t)$ to give

$$\tau_n^{(j)} = -\sum_{m=0}^{n-1} \int_0^\infty dt\, t \frac{dP_m^{(j)}(t)}{dt} \\ = \sum_{m=0}^{n-1} \int_0^\infty dt\, P_m^{(j)}(t). \tag{S20}$$

This model is valid for arbitrary epidemic dynamics in the origin (via the appropriate specification of $\Lambda_j(t)$ in Eq. S16). We now focus on an important specific case, where the epidemic is growing exponentially in the origin location.

B. Exponential growth in the origin. Assuming an exponentially growing epidemic in the origin, $I_i(t) = I_i(0)e^{\alpha_i t}$ then it can then be shown that (see e.g. (8) and (9)),

$$\int_0^\infty dt\, P_m^{(j)}(t) = \frac{1}{\alpha_i} e^{\kappa_j} E_{m+1}(\kappa_j), \tag{S21}$$

where $\kappa_j = h_j(0)/\alpha_i$. The special function $E_m(\kappa_j)$ is the exponential integral,

$$E_m(\kappa_j) = \int_1^\infty ds\, \frac{e^{-\kappa_j s}}{s^m}. \tag{S22}$$

Substituting Eqs. S21 into Eq. S20 gives the expected time of the n-th importation,

$$\tau_n^{(j)} = \frac{1}{\alpha_i} e^{\kappa_j} \sum_{m=1}^{n} E_m(\kappa_j). \tag{S23}$$

The expected time between successive importations (i.e. the expected inter-arrival time) is

$$\tau_n^{(j)} - \tau_{n-1}^{(j)} = \frac{1}{\alpha_i} e^{\kappa_j} E_n(\kappa_j) \text{ for } n = 2, 3, 4 \ldots. \tag{S24}$$

Assuming a small latent period relative to infectious period, and that incidence of variant is the exponent α_j can be expressed in terms of the variant reproductive number and other epidemiological parameters as (1)

$$\alpha_i \approx (R_0 c_{i,i}(1 - \phi_i) - 1)\gamma. \tag{S25}$$

C. Upper bound on the expected time of importation. While the exponential integral is exact, as a special function it makes it difficult to intuit the dependence of $\tau_n^{(j)}$ on the epidemiological parameters that compose κ_j and α_i. We therefore derived an upper bound on $\tau_n^{(j)}$ by making use of Jensen's inequality. To do this, we first use Eqs. S16 and S19 to re-write the expression for the expected time of the n-th importation in terms of the cumulative hazard $\Lambda_j(t)$,

$$\tau_n^{(j)} = \int_0^\infty dt\, t f_n^{(j)}(t) \\ = \int_0^\infty dt\, t h_j(t) \frac{\Lambda_j(t)^{n-1}}{(n-1)!} e^{-\Lambda_j(t)} \\ = \int_0^\infty dt\, t \frac{d\Lambda_j(t)}{dt} \frac{\Lambda_j(t)^{n-1}}{(n-1)!} e^{-\Lambda_j(t)}. \tag{S26}$$

If $\Lambda_j(t)$ is invertible (this is satisfied if $\Lambda_j(t)$ is strictly monotonic increasing, i.e $h_j(t) > 0$ for all t) then

$$\tau_n^{(j)} = \int_0^\infty du\, \Lambda_j^{-1}(u) \frac{u^{n-1}}{(n-1)!} e^{-u}, \tag{S27}$$

and we can identify $\frac{u^{n-1}}{(n-1)!}e^{-u}$ as the PDF of a gamma distributed random variable U with shape parameter n and scale parameter 1. It therefore follows that $\tau_n^{(j)} = \mathbb{E}[\Lambda_j^{-1}(U)]$.

If $\Lambda_j^{-1}(u)$ is a concave function (i.e. $\frac{dh_j(t)}{dt} > 0$; the case for exponentially growing epidemic) then $\mathbb{E}[\Lambda_j^{-1}(U)]$ satisfies Jensen's inequality, $\mathbb{E}[\Lambda_j^{-1}(U)] \leq \Lambda_j^{-1}(\mathbb{E}[U])$. From the properties of the gamma distribution we have $\mathbb{E}[U] = n$, therefore

$$\tau_n^{(j)} \leq \Lambda_j^{-1}(n). \tag{S28}$$

For the exponentially growing epidemic detailed in the previous section,

$$\tau_n^{(j)} \leq \frac{1}{\alpha_i}\ln(1 + n/\kappa_j). \tag{S29}$$

D. Statistical inference using the TIPP model. The probability of observing a sequence of N importations at arrival times $\{t_n\}_{n=1}^N = (t_1, t_2, \ldots, t_N)$ during an interval $[t_0, t_N]$ can be found by solving the master equation Eq. S17, and is

$$\begin{aligned} P(\{t_n\}_{n=1}^N) &= \prod_{n=1}^{N}\left(h_j(t_n)e^{-\int_{t_{n-1}}^{t_n} ds\, h_j(s)}\right) \\ &= \left(\prod_{n=1}^{N} h_j(t_n)\right) e^{-\int_{t_0}^{t_N} ds\, h_j(s)}. \end{aligned} \tag{S30}$$

If $h_j(t)$ can be written as a function of a set of model parameters, θ, then Eq. S30 can be used to perform likelihood-based inference. The log-likelihood is

$$\ell(\theta) = \sum_{n=1}^{N} \ln h_j(t_n) - \int_{t_0}^{t_N} ds\, h_j(s). \tag{S31}$$

If there is little change in the population immunity in the destination during $[t_0, t_N]$ then, from Eq. S15, $h_j(t) \approx q_j I_i(t)$, with $q_j = \beta c_{i,j}(1 - \phi_j)$. Given a time series of the number of individuals infectious with the variant in location i (the origin), a maximum likelihood estimate for q_j can be found by maximising Eq. S31 over q_j. Furthermore, by using this parameterisation, Eq. S31 can be written as

$$\begin{aligned} \ell(q_j) &= \sum_{n=1}^{N} \ln(q_j I_i(t_n)) - q_j \int_{t_0}^{t_N} ds\, I_i(t) \\ &= N \ln q_j + \sum_{n=1}^{N} \ln(I_i(t_n)) - q_j \int_{t_0}^{t_N} ds\, I_i(t). \end{aligned} \tag{S32}$$

This equation has a unique maximum,

$$q_j^* = \frac{N}{\Phi}, \tag{S33}$$

where $\Phi = \int_{t_0}^{t_N} ds\, I_i(t)$. Note that the parameters which compose q_j (i.e. β, $c_{i,j}$, ...) can not be individually identified with this model.

S3. Modelling the identification of variants

A. Including variant detections in the model and accounting for observation delays. Building on our stochastic transmission model, we derived a mathematical expression for the probability of making d observations of the variant in the origin location before it causes n imported infections in location j. We first present the derivation of our results for a general monitoring process, and then consider two specific data streams: random infection sequencing data and mortality data.

We assume that the monitoring effort captures a fraction of variant infections, p_O. We can then decompose the total number of variant infections in the origin $Q_i(t)$ into a sum of two counting processes, $Q_i(t) = O_i(t) + U_i(t)$, where $O_i(t)$ is the number of infections that are ultimately detected,

$$O_i(t) = Y_i^O\left(\int_0^t ds\, p_O \lambda_i(s)(1 - \phi_i(s))N_i\right), \tag{S34}$$

and $U_i(t)$ are the numbers of infections that are ultimately not detected. As above, we assume that emergence of the variant takes place over a fast timescale such that $\phi_i(t)$ does not appreciably change (e.g. due to vaccine administration or infection).

Crucially, $O_i(t)$ counts the number of individuals exposed at time t that are *ultimately detected*, and not the number of variant infections detected up to time t, which we denote $D_i(t)$. Regardless of detection method, these two will not be equivalent due to the lag between exposure and detection. By assuming that incidence in the origin is sufficiently large to be well approximated by the deterministic dynamics (Eqs. S7–S11), we can include observation delays in our model (see e.g. (10)), and derive an expression for $D_i(t)$,

$$D_i(t) = Y_i^D\left(\int_0^t ds \int_0^s ds'\, p_O\beta c_{i,i}(1-\phi_i)K(s-s')I_i(s')\right), \tag{S35}$$

where $K(s-s')$ is the distribution of delays between infection and observation.

B. Exponential growth in the origin. We now focus on the specific case where variant incidence is growing exponentially in the origin, such that $I_i(t) = I_i(0)e^{\alpha_i t}$. After substituting this expression into Eq. S35 we have,

$$D_i(t) = Y_i^D\left(\int_0^t ds \int_0^s ds'\, p_O\beta c_{i,i}(1-\phi_i)K(s-s')I_i(0)e^{\alpha_i s'}\right). \tag{S36}$$

Furthermore, if there has been sufficient spread in the origin such that $e^{\alpha_{i,a} t} \gg 1$ (necessary for the deterministic approximation), we can: i) approximate the integral over s' by extending the lower bound to $-\infty$ ii) perform a change of variables $s' \to s - s'$, giving

$$\begin{aligned} D_i(t) &\approx Y_i^D\left(\int_0^t ds \int_{-\infty}^s ds'\, p_O\beta c_i(1-\phi_i)K(s-s')I_i(0)e^{\alpha_i s'}\right) \\ &= Y_i^{(O)}\left(\int_0^t ds \int_0^\infty ds'\, p_O\beta c_{i,i}(1-\phi_i)K(s')I_i(0)e^{\alpha_i (s-s')}\right). \end{aligned} \tag{S37}$$

This approach allows us to deconvolve the integrals and isolate all terms that depend on s', and we define the function

$$m(\alpha_i) = \int_0^\infty ds'\, K(s')e^{-\alpha_i s'}. \tag{S38}$$

This function is the moment generating function of the observation delay distribution $K(\cdot)$, and satisfies $0 < m(\alpha_{i,a}) < 1$ for all $\alpha_{i,a} > 0$. Substituting Eq. S38 into Eq. S37 and using $I_i(t) = I_i(0)e^{\alpha_i t}$ we find that

$$D_i(t) = Y_i^D\left(p_O\beta c_{i,i}(1-\phi_i)m(\alpha_i)\int_0^t ds\, I_i(s)\right). \tag{S39}$$

Finally, we define the local variant effective reproductive number, $R_{i,i} = \beta c_{i,i}(1-\phi_i)/\gamma$ and the cumulative variant infections $\chi_i(t) = \int_0^t ds\, \gamma I_i(s)$ (see e.g. (1)) to give

$$D_i(t) = Y_i^D\Big(p_O m(\alpha_i)R_{i,i}\chi_i(t)\Big). \tag{S40}$$

We see that the expected number of detected infections is proportional to the cumulative number of infections, with the proportionality determined by: i) the detection probability, ii) the local effective reproductive number and iii) the combined effects of the observation delay and epidemic growth rate. We see that the effect of the observation delay is to introduce a “discounting factor", $m(\alpha_i)$, that shrinks the Poisson process, due to the dependence of the observations on the size of the epidemic at earlier time, rather than just the size at time t, i.e. $\chi_i(t)$. The magnitude of the shrinkage depends on the statistical properties of the delay distribution (e.g. the mean delay)and the growth rate of the variant in the origin, α_i. For a given observation delay distribution and parameterization, faster epidemic growth corresponds to larger shrinkage.

C. Quantifying the prospects of identifying a variant before exportation. Having derived expressions for the number of detections up to time t, $D_i(t)$ (see Eq. S40) and the number of exported infections $Q_j(t)$ (see Eqs. S14 and S15), we used these results to derive the probability of identifying a novel variant before infections are exported.

We start our derivation by defining the total number of events, $\Psi(t) = D_i(t) + Q_j(t)$, where an event is either variant infection detection or exportation. From Eqs. S14, S15 and S40, we find that $\Psi(t)$ is a superposition of two TIPPs,

$$\begin{aligned} \Psi(t) &= Y_i^D\Big(p_O m(\alpha_i)R_{i,i}\chi_i(t)\Big) + Y_j^Q\Big(R_{i,j}\chi_i(t)\Big) \\ &= Y_i^\Psi\Big(\big(p_O m(\alpha_i)R_{i,i} + R_{i,j}\big)\chi_i(t)\Big), \end{aligned} \tag{S41}$$

where we have defined the inter-location effective reproductive number $R_{i,j} = \beta c_{i,j}(1-\phi_j)/\gamma$ and again make use of the cumulative variant infections $\chi_i(t) = \int_0^t ds\, \gamma I_i(s)$. Conditional on $\Psi(t) = \psi$ events occurring, the number of events which are detections, d, is binomially distributed,

$$P\big(D_i(t) = d | \Psi(t) = \psi\big) = \binom{\psi}{d} q^d (1-q)^{\psi-d} \text{ for } d = 0, \ldots, \psi, \tag{S42}$$

with

$$q = \frac{p_O m(\alpha_i) R_{i,i}}{p_O m(\alpha_i) R_{i,i} + R_{i,j}}. \tag{S43}$$

Crucially, as the underlying processes both depend on time via $\chi_i(t)$, the binomial distribution in Eq. S42 is independent of time. From Eq. S42, we see that the number of exportations before d variant infections are detected, n, is negatively binomially distributed with probability mass function

$$f(n; d, q) = \binom{n+d-1}{d-1} (1-q)^n q^d, \tag{S44}$$

where q is defined in Eq. S43.

If d detections are necessary for the variant to be identified, then the probability that the variant is identified before any infections are exported, P_0, is given by $f(0; d, q)$. In terms of the underlying epidemiological parameters,

$$P_0 = \left(\frac{p_O m(\alpha_i) R_{i,i}}{p_O m(\alpha_i) R_{i,i} + R_{i,j}}\right)^d. \tag{S45}$$

By rearranging Eq. S45, we can derive an expression for the proportion of infections that must be detected for a given P_0 to be achieved,

$$p_O = \frac{R_{i,j} P_0^{1/d}}{m(\alpha_i) R_{i,i}(1 - P_0^{1/d})}. \tag{S46}$$

D. Identifying variant using sequencing data. For identifying a variant in randomly sampled sequencing data, the detection probability p_O is just equal to p_S, the proportion of infections that are randomly sequenced. The observation lag is a composite of i) the lag between exposure and sequencing (likely only after symptom onset), ii) the sequence processing time between sequencing and availability of results and iii) the lag between sequencing results and their dissemination. Due to a lack of detailed data on the distributions of each of these lags, we assumed a fixed delay τ_S, i.e. $K(t) = \delta(t - \tau_S)$. The observation lag discount factor is $m_S(\alpha_i) = e^{-\alpha_i \tau_S}$. We considered a range of plausible values for both p_S and τ_S (see main text). The sequencing probability necessary to achieve a given identification probability is (assuming $c_{i,j} \ll 1$)

$$p_S \approx \frac{c_{i,j} P_0^{1/d_S}}{(1 - P_0^{1/d_S}) e^{-\alpha_i \tau_S}}. \tag{S47}$$

E. Identifying a variant using mortality data. In a study of Australian COVID-19 deaths, the distribution of time of symptom onset was estimated using gamma distribution (11) with shape parameter $a = 2.55$ and scale parameter $\theta = 7.10$ (corresponding to a mean delay of 18.1 days and a standard deviation of 11.3 days). We combined this with with a delay between exposure and symptom onset of $\tau_{\text{SO}} = 6.5$ days (12). The resulting observation lag discount factor is $m_M(\alpha_i) = (1 + \theta \alpha_i)^{-a} e^{-\alpha_i \tau_{\text{SO}}}$. For mortality data the detection probability is equal to the infection fatality probability. We assumed that the variant had an infection fatality probability $p_\mu = 0.01$, comparable with estimates for the original variant early in the pandemic (13).

F. Identifying a variant using vaccine-breakthrough mortality data. Identifying a variant based on vaccine-breakthrough mortality data involves only counting deaths in vaccinated individuals. The derivation proceeds along identical lines as for general mortality data (see above), however with one difference. The probability of observation, p_O, is the product of: i) the fraction of infections that are vaccinated individuals, f_V, and ii) the infection fatality probability for vaccinated individuals, p_μ^V.

The fraction of infections that are in vaccinated individuals can be calculated by splitting up the $E_i(t)$ and $I_i(t)$ compartments in the dynamical equations (Eqs. S7–S11) to explicitly indicate whether the individual was previously in the S, R or V compartment. The fraction $f_V(t)$ is then given by

$$f_V(t) = I_i^V(t) / (I_i^S(t) + I_i^R(t) + I_i^V(t)). \tag{S48}$$

For an epidemic that is exponentially growing in the origin, the dynamics quickly collapse onto the eigendirection of the largest eigenvalue (see e.g. (14)). From this time-scale separation analysis, the fraction of unvaccinated infections over time is approximately constant,

$$f_V(t) = \frac{(1-\varepsilon_V)V_i}{(1-\phi_i)}. \tag{S49}$$

The observation probability is then $p_O = p_\mu^V(1-\varepsilon_V)V_i/(1-\phi_i)$

In Figure S2 we plot results for varying vaccine coverage, v, and protective efficacy ε_V, assuming (to simplify the analysis) that the immune profile in both origin and destination is the same (i.e. $\phi_i = \phi_j$). In particular, we see that the probability there is at least one exported infection is a non-trivial function of these two parameters (Figure S2D).

S4. Combining monitoring schemes

Along similar lines to Section C, we can extend our model to quantify the prospects of identifying a variant before exportation when multiple monitoring schemes are employed. Furthermore, we can use this model to evaluate which detection method is more likely to identify the variant first. In this section we consider two monitoring approach running in parallel, one using sequencing data (subscripts S) and the other mortality data (subscripts M).

The derivation follows similar lines to the derivation for one monitoring scheme in SI Sec. C. We define the total number of events to be the sum of the number of detections with each monitoring method and the number of exported infections, i.e.$\Psi = D_S(t) + D_A(t) + Q_j(t)$, which is a superposition of three TIPPs,

$$\begin{aligned}\Psi(t) &= Y_i^S\Big(p_S m_S(\alpha_i)R_{i,i}\chi_i(t)\Big) + Y_i^M\Big(p_M m_M(\alpha_i)R_{i,i}\chi_i(t)\Big) + Y_j^Q\Big(R_{i,j}\chi_i(t)\Big)\\ &= Y_i^\Psi\Big(\big(\kappa_S + \kappa_M + \kappa_E\big)\chi_i(t)\Big),\end{aligned} \tag{S50}$$

where, to simplify the notation, we use $\kappa_S = p_S m_S(\alpha_i)R_{i,i}$, $\kappa_M = p_M m_M(\alpha_i)R_{i,i}$ and $\kappa_Q = R_{i,j}$. As before, $\chi_i(t) = \int_0^t ds\,\gamma I_i(s)$.

Conditioned on $\Psi(t) = \psi$ events, the numbers of detections and importations follows a multinomial distribution, with probability mass function $P(D_S = x, D_M = y, Q_j = n|\Psi = \psi) = b(x, y, n; \psi, \pi_S, \pi_M)$ given by

$$b(x, y, n; \psi, \pi_S, \pi_M) = \begin{cases} \frac{(\psi!}{x!y!n!}\pi_S^x\pi_M^y(1-\pi_S-\pi_M)^n & \text{for } x+y+n=\psi,\\ 0 & \text{otherwise,}\end{cases} \tag{S51}$$

where $\pi_a = \kappa_a/(\kappa_S + \kappa_M + \kappa_Q)$ for $a = S$ and M. As was the case when considering only one monitoring scheme, Eq. S51 does not depend the cumulative incidence, $\chi_i(t)$. We now focus on the probability that there are y detected variant deaths and n exported infections before there are x detected variant sequences, $P(D_M = y, Q_j = n|D_S = x)$. From Eq. S51, it can be seen this probability follows a negative multinomial distribution, with probability mass function $P(D_M = y, Q_j = n|D_S = x) = g(y, n; x, \pi_S, \pi_M)$, given by

$$\begin{aligned}g(y, n; x, \pi_S, \pi_M) &= \pi_x b(x-1, y, n; x+y+n-1, \pi_S, \pi_M)\\ &= \frac{(x+y+n-1)!}{(x-1)!y!n!}\pi_S^x\pi_M^y(1-\pi_S-\pi_M)^n.\end{aligned} \tag{S52}$$

By defining the probability $q_S = \pi_S/(1-\pi_M)$, we can write Eq. S52 as

$$g(y, n; x, \pi_S, \pi_M) = \frac{(x+n-1)!}{(x-1)!n!}q_S^x(1-q_S)^n\frac{(x+y+n-1)!}{(x+n-1)!y!}\pi_M^y(1-\pi_M)^{x+n}, \tag{S53}$$

where we use that $1-q_S = (1-\pi_S-\pi_M)/(1-\pi_M)$. This equation can be recognised as the product of two negative binomial probability mass functions,

$$g(y, n; x, \pi_S, \pi_M) = f(n; x, q_S)f(y; n+x, 1-\pi_M). \tag{S54}$$

While other factorizations are possible, this is the most useful for our purposes (as the only dependence on y is in the second function). This factorization also elucidates the structure of $g(y, n; x, \pi_S, \pi_M)$, namely that it is the product of two terms: i) the probability that there are n exports given x sequencing detections, $f(n; x, q_S)$, and ii) the probability that there are y mortality detections given that there are a combined $x+y$ exported infections and sequencing detections, $f(y; n+x, 1-\pi_M)$.

The probability of identifying the variant first in sequencing data before i) it is identified in mortality data and ii) infection is exported, P_S can be found from Eq. S54 by setting $n = 0$, $x = d_S$ (the sequencing detection threshold) and summing over $y = 0, \ldots d_M - 1$ (i.e. total mortality counts that are less than the mortality detection threshold). Explicitly, we have

$$\begin{aligned}P_S &= f(0; d_S, q_S)\sum_{y=0}^{d_M-1} f(y; d_S, 1-\pi_M)\\ &= q_S^{d_S} I_{1-\pi_M}(d_S, d_M)\end{aligned} \tag{S55}$$

where $I_{1-\pi_M}(d_S, d_M)$ is the regularized incomplete beta function (9). The equivalent result for identifying the variant first in mortality data, $P_M = q_M^{d_M} I_{1-\pi_S}(d_M, d_S)$, can be trivially found by repeating the derivation with S and M exchanged. Finally, the total probability of identifying the variant before the exportation of infection, regardless of the method that identifies the variant, is simply

$$P_0 = P_S + P_M. \tag{S56}$$

S5. Identification of variant through clusters of incidence

In the previous section we explored prospects for identifying a novel variant used random sequencing data and mortality data. If it emerges during a period of low SARS-CoV-2, it is possible for a novel variant the be identified through clusters of incidence directly. To adapt our methodology to this monitoring approach, we assumed that the variant is identified after the cluster of detected infections reaches d_C cases and that this threshold is reasonably large ($d_C \gtrsim 1000$), meaning that the effects of demographic stochasticity on the growth in detected cases is small. Furthermore, we assume that incidence in the cluster of detected cases grows at the same rate as overall incidence of the variant in the origin (i.e. there is no difference in reproductive number between individuals in the cluster and the wider population). Given these assumptions, the proportion of total variant infections that are detected is a constant over time, which we denote by p_C. Additionally, the cumulative number of detected cases in the cluster, $D_i^C(t)$ has a similar mathematical structure to the general monitoring case (see Sec. S3, Eq. S35). The key difference is that, because we assumed the effects of demographic stochasticity were small, $D_i^C(t)$ is instead a deterministic process (cf. Eq. S35). Making the same assumption of exponential growth in the origin as above, $I_i(t) = I_i(0)e^{\alpha_i t}$, we find that

$$\begin{aligned} D_i^C(t) &= \int_0^t ds \int_0^s ds'\, p_C \gamma R_{i,i} K(s-s') I_i(s') \\ &\approx \int_0^t ds p_C \gamma R_{i,i} m_C(\alpha_i) I_i(s') \\ &= p_C R_{i,i} m_C(\alpha_i) \chi_i(t), \end{aligned} \tag{S57}$$

with $R_{i,i} = R_0 c_{i,i}(1-\phi_i)$, $m(\alpha_i) = \int_0^\infty ds'\, K(s')e^{-\alpha_i s'}$ and $\chi_i(t) = \int_0^t ds\, \gamma I_i(s)$, as defined above. For this monitoring approach, the delay distribution $K(t)$ corresponds to the lag between exposure and case confirmation via testing (accounting for the combined delays between exposure, symptom onset and test seeking and also test processing times).

Since $D_i^C(t)$ is a strictly increasing deterministic function, the time of variant identification, t_D, is the unique time that solves $D_i^C(t_D) = d_C$. By rearranging Eq. S57, we find that the cumulative number of variant infections at the time of identification is given by

$$\chi_i(t_D) = \frac{d_C}{p_C R_{i,i} m_C(\alpha_i)}. \tag{S58}$$

As with the general monitoring case in Sec. S3, the the number of imported infections in the destination through time, $Q_j(t)$, is given by Eqs. S14 and S15. At the time of identification this is equal to

$$\begin{aligned} Q_j(t_D) &= Y_j^Q \left(\int_0^t ds \gamma R_{i,j} I_i(s') \right) \\ &= Y_j^Q \left(R_{i,j} \chi_i(t_D) \right), \end{aligned} \tag{S59}$$

where $R_{i,j} = R_0 c_{i,j}(1-\phi_j)$ (see Sec. C). Finally, after substituting in for $\chi_i(t_D)$, we find that

$$Q_j(t_D) = Y_j^Q \left(\frac{d_C R_{i,j}}{p_C R_{i,i} m_C(\alpha_i)} \right). \tag{S60}$$

Eq. S60 implies that the number of imported infections at the time of identification, n, is Poisson distributed, with expectation value $\lambda_C = \frac{d_C R_{i,j}}{p_C R_{i,i} m_C(\alpha_i)}$. The probability that no infections are imported before identification is $P_C = e^{-\lambda_C}$.

This result is in contrast with the result for the previous monitoring methods, which was that n is negatively binomally distributed, with probability mass function $f(n; d, q)$ (see Eq. S44). This difference in resultant distribution stems from the assumption for the incidence cluster method that the detection of cases is approximately deterministic, whereas for the other two methods the detection of cases is stochastic. Indeed, if we assume that a large number of sequences are necessary for identification using random case sequencing, and that the per infection rate of sequencing, $\kappa_S = p_S m_S(\alpha_i) R_{i,i}$, is much larger than the per infection rate of importation $R_{i,j}$ then the negative binomal distribution in Eq. S44 can be approximated by a Poisson distribution. This can shown starting from Eq. S43, which can be approximated as

$$q \approx 1 - R_{i,j}/p_S m_S(\alpha_i) R_{i,i}. \tag{S61}$$

Consequently, as $d_S \gg n$, Eq. S44 is approximately

$$\begin{aligned} f(n; d_S, q) &= \binom{n + d_S - 1}{d_S - 1} (1-q)^n q^{d_S} \\ &\approx \frac{1}{n!} \left(\lambda_S\right)^n e^{-\lambda_S}, \end{aligned} \qquad \text{[S62]}$$

where $\lambda_S = \frac{d_S R_{i,j}}{p_S m_S(\alpha_i) R_{i,i}}$. This is the probability mass function of a Poisson distributed random variable with a rate parameter λ_S which has the same structure in terms of the underling model parameters as λ_C.

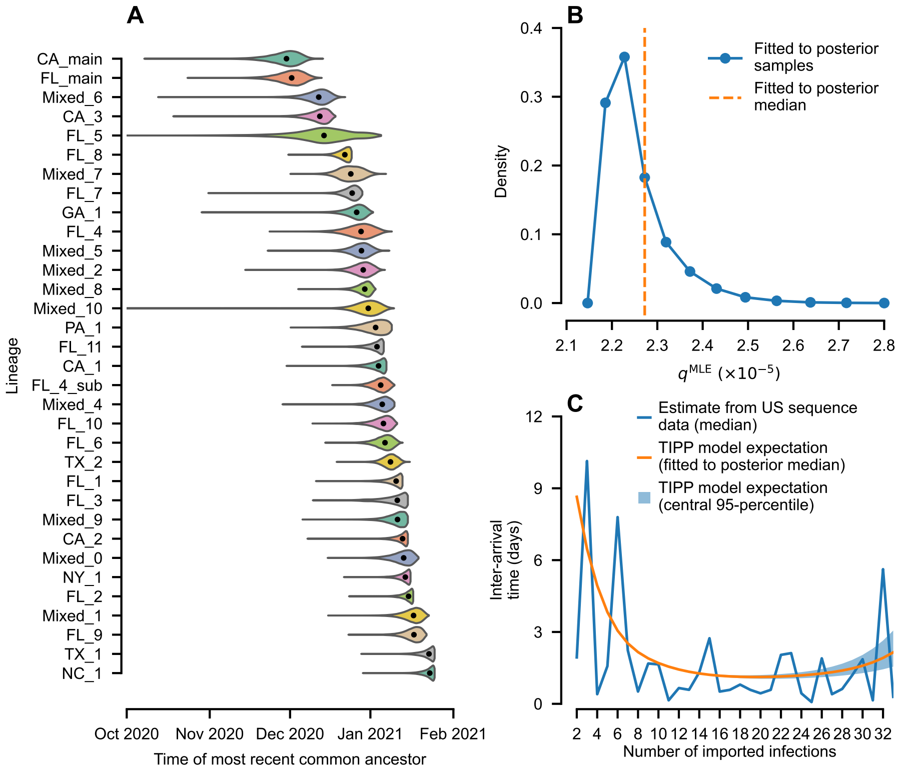

Fig. S1. Sensitivity of the estimated expected inter-arrival time to uncertainty stemming from the underlying phylogenetic analysis of alpha variant importation in the US (15). A) Violin plots showing the posterior density for the time of most recent common ancestor (TMRCA) from the MCMC analysis of sequence data presented in (15). The TMRCA is treated as a proxy for the time the lineage was imported into the US (15). Black dots indicate the posterior median TMRCA. B) Distribution of maximum likelihood estimates (MLEs) of the parameter q_j, found by fitting to each posterior sample individually. The MLE found by fitting to the posterior median (as shown in Figure 2) is indicated by the vertical dashed line. The maxima fall on discrete values due to the time-series of individuals infectious with the alpha variant in the UK (a covariate in the model, see Section S2) being constant within each day. C) Uncertainty in the estimated expected inter-arrival time. Due to the limited range of estimates of q^{MLE} (due in turn to the limited range in estimates of the TMRCA for the final imported case, see Eq. S33), the central 95-percentile for the distribution in the expected inter-arrival time is extremely narrow across the series of imported infections. For context, the median estimate of the observed inter-arrival time is also shown (blue line).

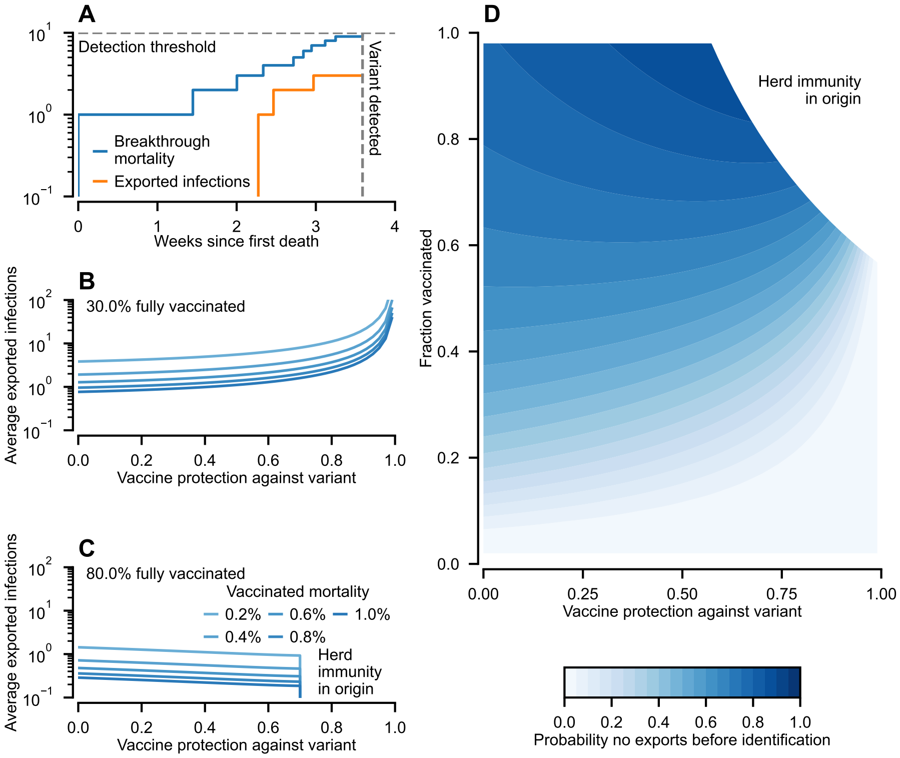

Fig. S2. Prospects for detecting and containing a variant with increased vaccine breakthrough mortality probability. A) Regardless of methodology, detection of the variant requires sufficient mortality for the variant to be detected (blue line). During that period, spillover transmission from the origin location may occur (orange line). B) The average exported infections prior to detection is inversely proportional to the probability of mortality from vaccine breakthrough infection, p_μ^V. Furthermore, assuming equal vaccine coverage in both the origin and destination location, at low-to-intermediate vaccine coverage (30% coverage shown), increased protection against breakthrough infection (ϵ_V) decreases the ability to detect the variant before spatial spread: vaccination protects against infection, reducing breakthrough mortality, while the large susceptible pool allows for incidence to increase – thereby increasing the probability of exportation. C) This pattern is reversed at high vaccine coverage: here, the protection against infection in the destination and the diminished susceptible pool in the origin both reduce the probability of exportation, and counteract the reduction in detectability of the variant (shown at 80% coverage). D) Heat map summarising the probability that the variant is identified before any infections are exported, as a function of protection against breakthrough infection and vaccine coverage assuming the probability of mortality from a vaccine breakthrough infection is 1%. For all panels in this figure we used the parameters $R_0 = 2.3$, $d_M = 10$, $c_{i,j} = 10^{-4}$ and assumed no natural immunity to variant infection, $\epsilon_R = 0$.

References

1. MJ Keeling, P Rohani, *Modeling Infectious Diseases in Humans and Animals.* (Princeton University Press), (2011).
2. TC Jones, et al., Estimating infectiousness throughout SARS-CoV-2 infection course. *Science* **373**, eabi5273 (2021).
3. Q Li, et al., Early transmission dynamics in Wuhan, China, of novel coronavirus-infected pneumonia. *The New Engl. J. Medicine* **382**, 1199–1207 (2020).
4. TG Kurtz, Strong approximation theorems for density dependent Markov chains. *Stoch. Process. their Appl.* **6**, 223–240 (1978).
5. NG Van Kampen, *Stochastic Processes in Physics and Chemistry.* (Elsevier) Vol. 1, (1992).
6. DF Anderson, TG Kurtz, *Stochastic Analysis of Biochemical Systems.* (Springer) Vol. 674, (2015).
7. Office of National Statistics, Population estimates for the UK, England and Wales, Scotland and Northern Ireland: mid-2020 (2020).
8. L Wang, JT Wu, Characterizing the dynamics underlying global spread of epidemics. *Nat. Commun.* **9**, 1–11 (2018).
9. NM Temme, *Special Functions: An Introduction to The Classical Functions of Mathematical Physics.* (John Wiley & Sons), (1996).
10. T Brett, T Galla, Stochastic processes with distributed delays: chemical langevin equation and linear-noise approximation. *Phys. Rev. Lett.* **110**, 250601 (2013).
11. IC Marschner, Estimating age-specific COVID-19 fatality risk and time to death by comparing population diagnosis and death patterns: Australian data. *BMC Med. Res. Methodol.* **21**, 1–10 (2021).
12. M Alene, et al., Serial interval and incubation period of COVID-19: a systematic review and meta-analysis. *BMC Infect. Dis.* **21**, 1–9 (2021).
13. A Hauser, et al., Estimation of SARS-CoV-2 mortality during the early stages of an epidemic: A modeling study in Hubei, China, and six regions in Europe. *PLoS Medicine* **17**, e1003189 (2020).
14. GW Constable, AJ McKane, T Rogers, Stochastic dynamics on slow manifolds. *J. Phys. A: Math. Theor.* **46**, 295002 (2013).
15. NL Washington, et al., Emergence and rapid transmission of SARS-CoV-2 B. 1.1. 7 in the United States. *Cell* **184**, 2587–2594 (2021).
