# Supplementary figures and images for: Containing novel SARS-CoV-2 variants at source is possible with high-intensity sequencing
Source: PNAS Nexus. 2022 Aug 19;1(4):pgac159. doi: 10.1093/pnasnexus/pgac159 (PMC9465520; doi:10.1093/pnasnexus/pgac159)

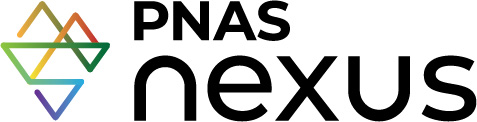

Supplement: pgac159_Supplemental_Files [file pgac159_supplemental_files.zip › PNASNEXUS-PNASNEXUS-2022-00266-T-s06.jpg]
